# Surgical correction of aberrant conjunctival overgrowth in a rabbit: a case report

**DOI:** 10.1186/2046-0481-66-18

**Published:** 2013-10-05

**Authors:** Joon Young Kim, David L Williams, Kyung-soo Rho, Kyung-hee Kim, Young-sun Lee, Soon-wuk Jeong

**Affiliations:** 1 Woo Sung Animal Medical Centre, 745-6 Banpo 1-dong, Seocho-gu 137-810, Seoul, Korea; 2 Department of Veterinary Medicine, Madingley Road, CB3 0ES Cambridge, United Kingdom; 3 Korea Animal Hospital, 527-3 Dogok Apt. Dogok 2-dong, Gangnam-gu 135-506, Seoul, Korea; 4 College of Veterinary Medicine, Kon Kuk University, 143-701 Seoul, Korea

**Keywords:** Aberrant conjunctival stricture, Conjunctival overgrowth, Circumferential conjunctival hyperplasia and plication, Epicorneal conjunctival membranes, Pseudopterygium

## Abstract

A dwarf rabbit presented with unilateral aberrant conjunctival growth. Allgoewer’s U-suture therapy was initially used to correct the overgrowth. Centrifugal incisions extending up to the limbus were made on the hypertrophic conjunctiva. Transpalpebral limbal fixation was performed next. When the symptoms recurred 3 weeks later, a second operation was performed using the Lembert suture method instead. The overgrowing membrane was excised radically just posterior to the limbus. The conjunctiva was then sutured using the Lembert pattern. The rabbit recovered with no further complications.

## Background

Aberrant conjunctival overgrowth (stricture) is a unique and unusual eye disease [1,2] and has only been observed in dwarf rabbits. It appears as a pink, double-layered, vascular membrane that overlies the full 360 degrees of the corneal rim and extends centrally. This condition is poorly understood and appears to be unique to the rabbit [3]. The pink tissue is a conjunctival fold, which grows centripetally from the bulbar conjunctiva at the limbus and obscures the cornea to varying degrees [3]. It remains attached at the limbus, but the central fold of tissue moves freely over the corneal surface [3,4] without adhesions [1,4]. In cases of aberrant conjunctival overgrowth, only loose-to-moderately firm focal adhesions at the peripheral cornea have been reported [5]. Generally, eyes with overgrowth do not have symptoms of conjunctivitis, but in some cases, mild inflammation can occur. Corneal oedema may present at the central conjunctival opening. Aberrant conjunctival overgrowth differs from human pterygium [1,4] and from feline symblepharon. In these two conditions, conjunctival overgrowth is intimately attached to the cornea, and the conjunctiva replaces the corneal epithelium [1].

Aberrant conjunctival stricture generally occurs in adult animals [1,2]. Neither topical nor systemic medication is effective for membrane regression or for prevention of further growth [3]. Only surgical intervention can remove the membrane, but simple resection of the membrane back to the limbus is not sufficient, because the membrane regrows rapidly [3]. Therefore, after membrane removal, the cut edge is sutured to the bulbar conjunctiva and sclera just behind the limbus [3]. Allgoewer et al. [1] reported surgical success in 10 eyes of 6 dwarf rabbits operated using Stades’ U-suture therapy for aberrant conjunctival stricture and overgrowth. Another method is the Lembert suture method for aberrant conjunctival overgrowth. Although steroid and cyclosporine treatments are ineffective, topical cyclosporine ointment is generally used after surgical correction to reduce the risk of regrowth.

Here, we report the clinical presentation of aberrant conjunctival stricture and overgrowth and report the therapeutic efficacy of the U-suture and Lembert suture methods.

## Case presentation

### History and ophthalmic findings

**Figure 1:**
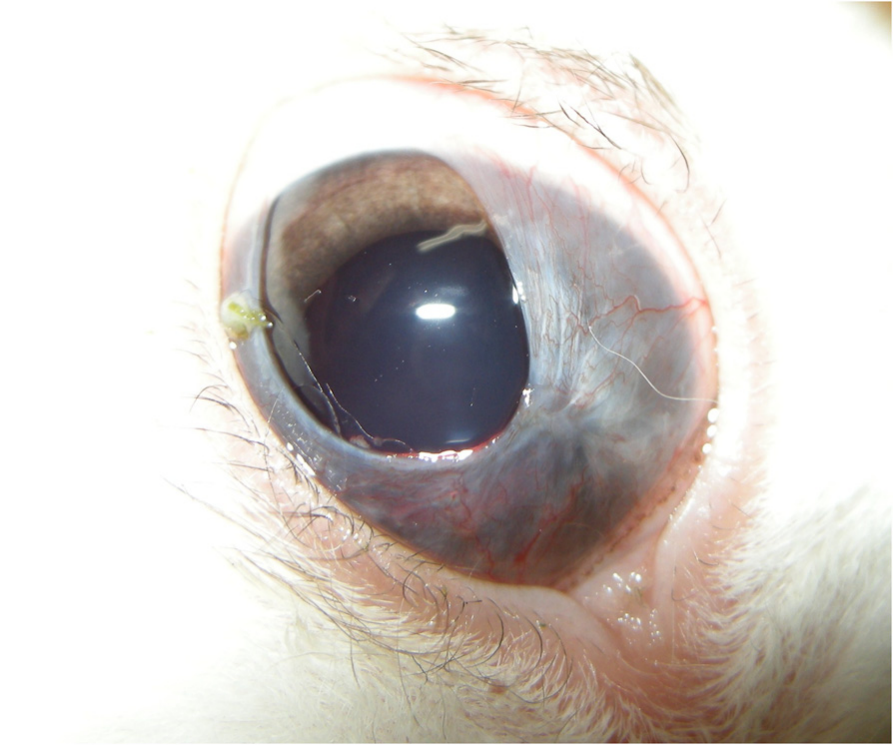
Aberrant conjunctival growth in the right eye.

**Figure 2:**
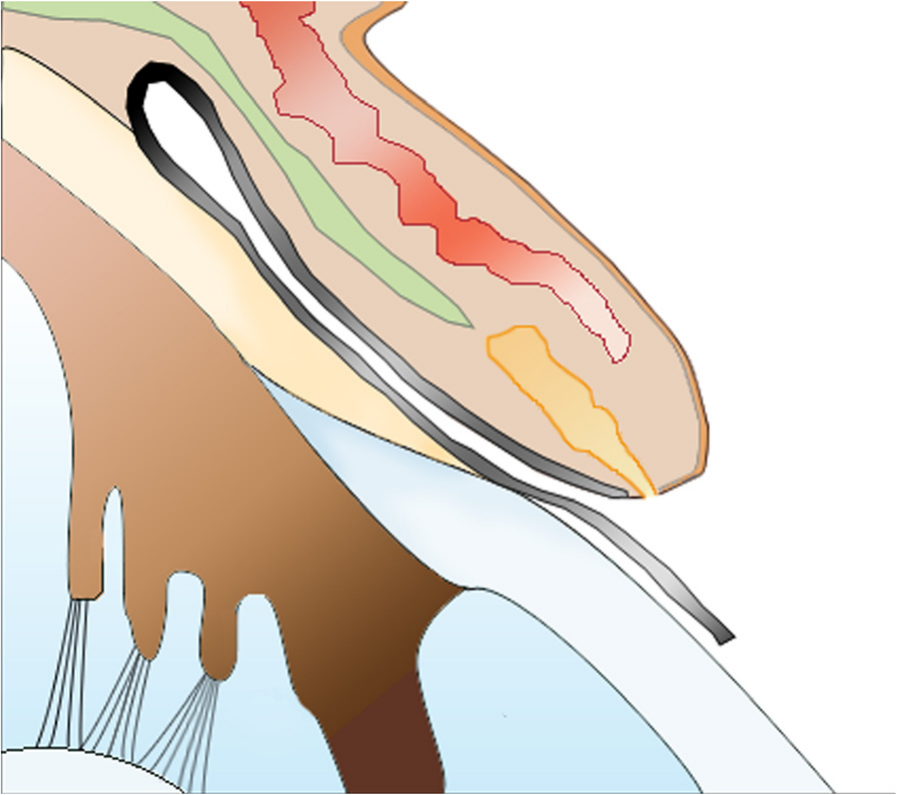
A conjunctival fold stretched over the cornea from the base of the tarsal plate (black line).

**Figure 3:**
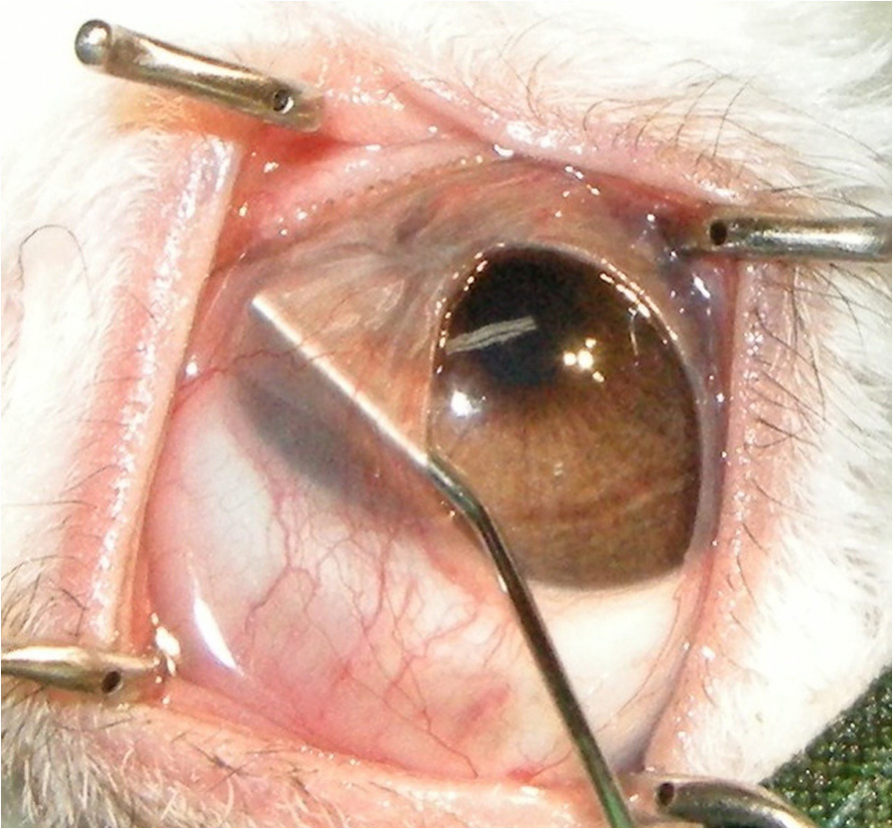
The conjunctival overgrowth did not adhere to the cornea.

A 10-month-old male dwarf rabbit presented to our clinic for a problem in the right eye. The eye had a pink, fleshy, vascular membrane covering 360° of the cornea and extending centrally. The membrane covered the entire cornea, except for the dorsolateral section (Figure 1). A conjunctival fold stretched over the cornea, originating at the limbus and inserting at the base of the tarsal plate of the lid margin (Figure 2). There were no corneal epithelium attachments, and the elastic membrane was easily lifted off the cornea (Figure 3). No other remarkable systemic or ophthalmic findings were noted.

### Surgical treatments and postoperative care

The conjunctival overgrowth was treated surgically using a modification of Stades’ U-suture technique as introduced by Allgoewer in [1]. After induction of anaesthesia by using propofol (10 mg/kg, Provive Inj 1%, Myungmoon Pharm. Co., LTD, Seoul, Korea), the patient was intubated. Maintenance was followed with isoflurane (Ifran, Hana Pharm. Co., LTD, Kyonggi-Do, Korea). After preparing the eyes with 0.2% povidone-iodine, the conjunctival fold was incised from the central rim up to its attachment at the limbus in 6 equally sized segments. All segments were then relocated to the normal position in the fornix and fixed into place with mattress sutures (Prolene 6–0) that passed through the skin [1].

**Figure 4:**
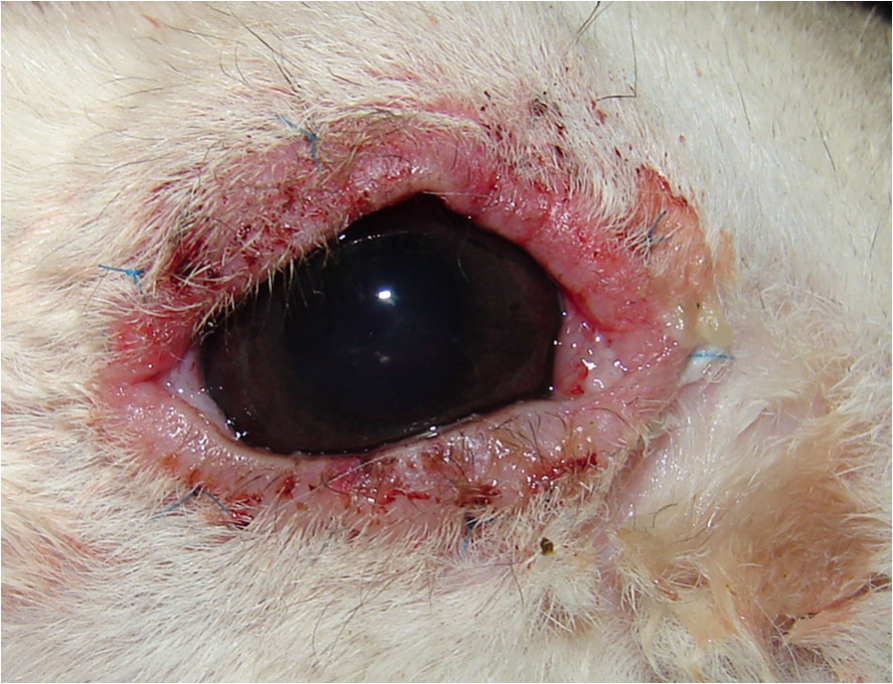
Postoperative appearance of the eye at 1 week after the first operation.

**Figure 5:**
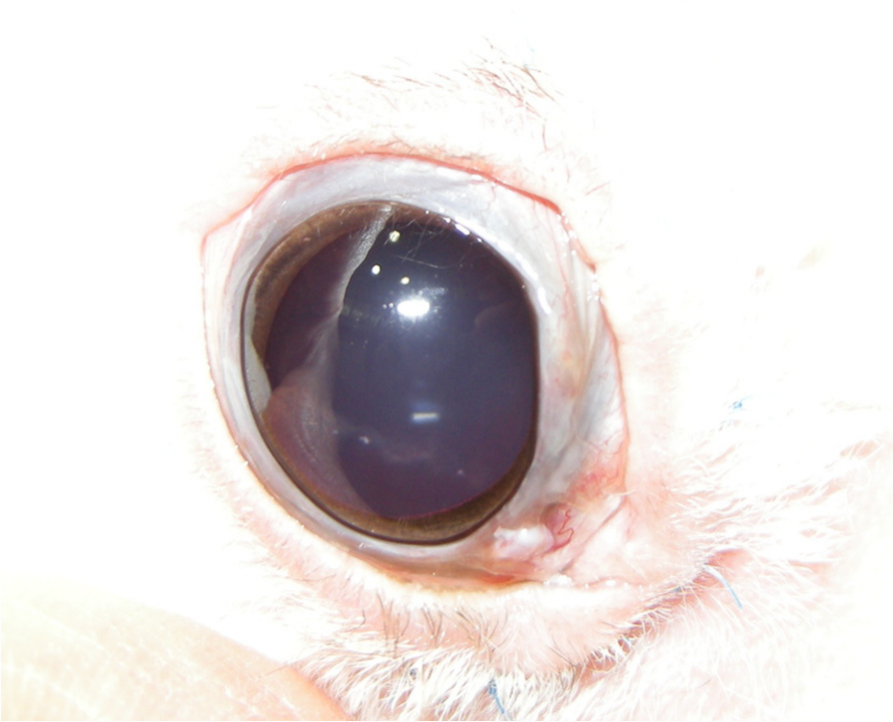
Postoperative appearance of the eye at 3 weeks after the first operation.

**Figure 6:**
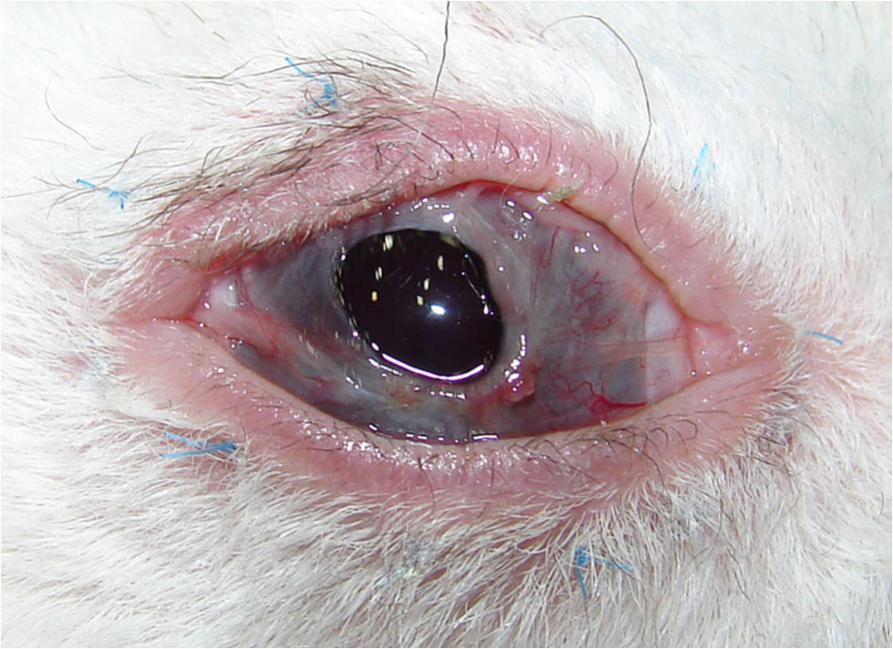
Postoperative appearance of the eye at 4 weeks after the first operation.

After surgery, neomycin, polymyxin B, and dexamethasone eye drops (Maxitrol®; Alcon-Couvreur, Puurs, Belgium) were applied twice a day for 3 weeks. One week after surgery, all eyelid surfaces exhibited moderate inflammation. Mild ocular discharge was noticed as well (Figure 4). The inflammation persisted despite the regular use of dexamethasone eye drops. Three weeks after surgery (Figure 5), inflammation had subsided, but the conjunctival fold began to regrow over the cornea. We then removed all sutures. Within the next week, the membrane had extended to cover the entire corneal surface, with the exception of a small central area (Figure 6).

**Figure 7:**
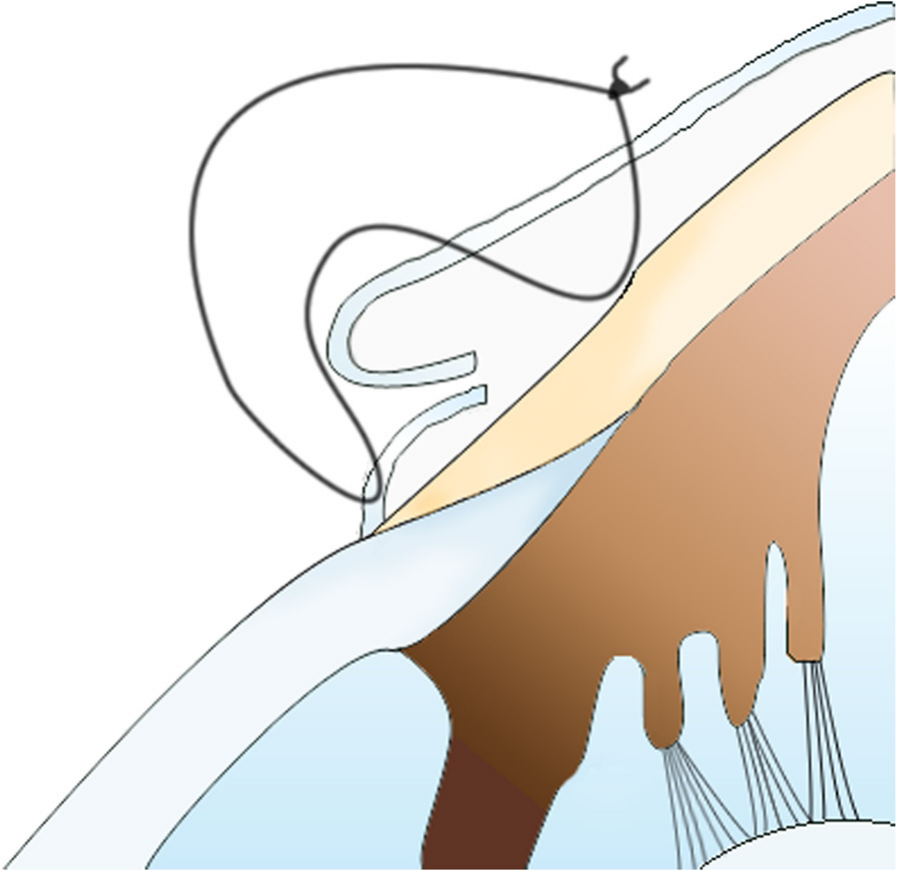
A diagram of the Lembert suture technique used to treat aberrant conjunctival overgrowth.

**Figure 8:**
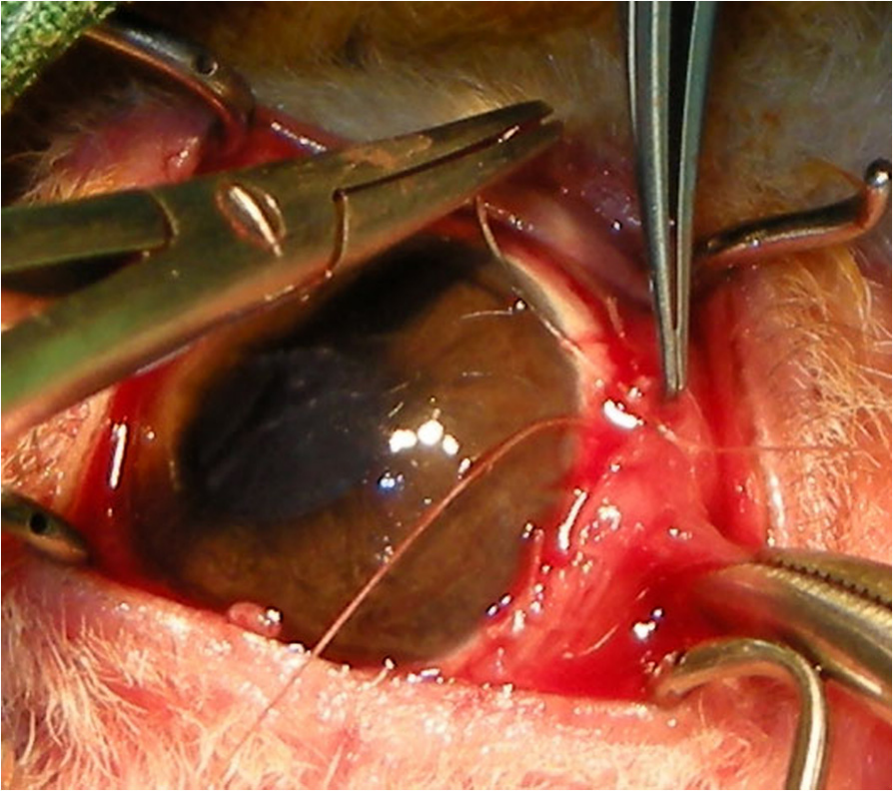
An intraoperative photo of the Lembert suture technique.

Five weeks after the first operation, we performed another operation, but this time, we used the Lembert suture technique (Figure 7). The surgical procedure used was otherwise similar to that reported by Turner [3]. After the induction of general anaesthesia, the membrane was simply resected back to the limbus. The entire membrane was removed, and the cut edges were inverted just behind the limbus by using the Lembert suture method (Vicryl 6–0) (Figures 7 and 8). Eight simple Lembert sutures were applied initially, followed by a continuous Lembert suture around the full limbal circumference (Vicryl 6–0) (Figures 7 and 8).

**Figure 9:**
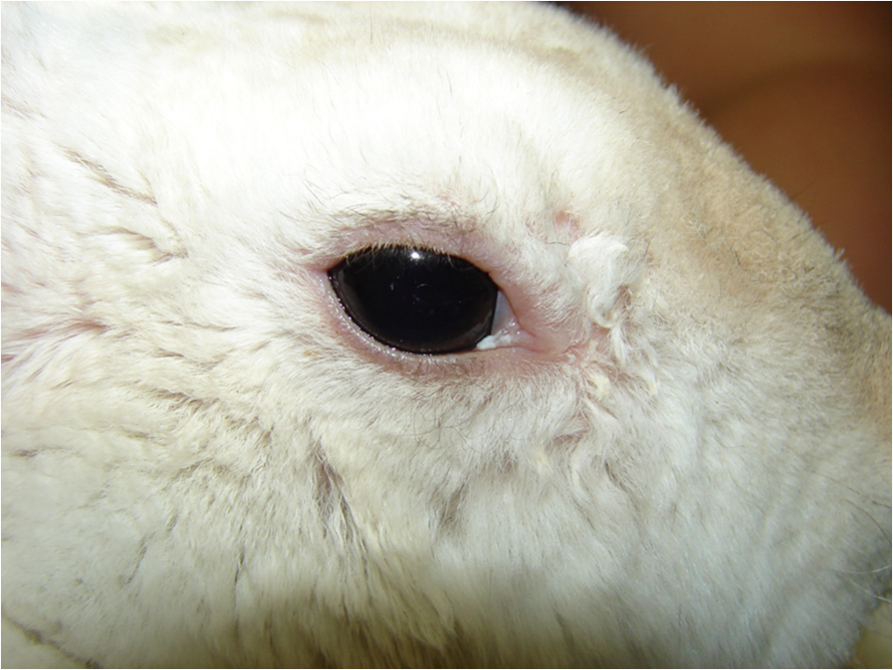
Four weeks after the second operation, no sign of eyelid inflammation or membrane regrowth is present.

Maxitrol® eye drops and cyclosporine ointment (0.2%, Optimmune®; Schering-Plough Animal Health, Heist-op-den-Berg, Belgium) were administered after the surgery. After surgery, no eyelid inflammation or ocular discharge was noted. Four weeks later, no sign of recurrence was present (Figure 9), and all medications were stopped. The rabbit was followed for an additional 11 months, but no signs of recurrence were detected.

## Conclusion

In the current case, we used Allgoewer’s method for the first operation. Moderate eyelid inflammation appeared postoperatively and persisted for 1 week. Three weeks after surgery, the conjunctival fold re-grew from the limbus. Within a week, the membrane had extended to cover most of the cornea. Despite the use of Maxitrol® eye drops, the eyelid and conjunctiva continued to exhibit signs of inflammation (Figure 5). To treat the recurrence, we used a different surgical method. The entire membrane was removed, and the cut edge was folded into the limbus, similar to the method introduced by Turner [3]. Eight simple Lembert sutures were created, followed by the placement of a continuous Lembert suture. With this method, the cut edge of the membrane is folded more securely under at the limbus.

When using Allgoewer’s method, the third eyelid caused some difficulty in suturing the tissue at the medial canthus. Furthermore, it was not easy to surgically handle six radial pieces of the membrane, which shrank after they were cut. After the first surgery, a certain degree of eyelid inflammation persisted, even with the use of steroid eye drops. This inflammation was not observed after the second surgery and may have occurred during the first surgery because the suture material (Prolene) can irritate the eyelid. Allgoewer et al. [1] did not report the same inflammation in their research.

## Competing interests

None of the authors of this paper has a financial or personal relationship with other organizations or people that could influence or bias the content of the paper.

## Authors’ contributions

JYK drafted the manuscript and helped to examine and manage the rabbit. DW helped to draft the manuscript. KSR, KHK and YSL helped with patient management. SWJ supervised patient management. All authors read and approved the final manuscript.
